# 3D-Printing and Machine Learning Control of Soft Ionic Polymer-Metal Composite Actuators

**DOI:** 10.1038/s41598-019-53570-y

**Published:** 2019-11-25

**Authors:** James D. Carrico, Tucker Hermans, Kwang J. Kim, Kam K. Leang

**Affiliations:** 1 University of Mary, School of Engineering, Bismarck, ND 58504 USA; 2 University of Utah, School of Computing, Utah Learning Lab for Manipulation Autonomy, University of Utah Robotics Center, Salt Lake City, UT 84112 USA; 3 University of Nevada, Las Vegas, Department of Mechanical Engineering, Active Materials and Smart Living (AMSL) Laboratory, Las Vegas, NV 89154 USA; 4 University of Utah, Department of Mechanical Engineering, Design Automation Robotics and Control (DARC) Lab, University of Utah Robotics Center, Salt Lake City, 84112 USA

**Keywords:** Mechanical engineering, Scientific data

## Abstract

This paper presents a new manufacturing and control paradigm for developing soft ionic polymer-metal composite (IPMC) actuators for soft robotics applications. First, an additive manufacturing method that exploits the fused-filament (3D printing) process is described to overcome challenges with existing methods of creating custom-shaped IPMC actuators. By working with ionomeric precursor material, the 3D-printing process enables the creation of 3D monolithic IPMC devices where ultimately integrated sensors and actuators can be achieved. Second, Bayesian optimization is used as a learning-based control approach to help mitigate complex time-varying dynamic effects in 3D-printed actuators. This approach overcomes the challenges with existing methods where complex models or continuous sensor feedback are needed. The manufacturing and control paradigm is applied to create and control the behavior of example actuators, and subsequently the actuator components are combined to create an example modular reconfigurable IPMC soft crawling robot to demonstrate feasibility. Two hypotheses related to the effectiveness of the machine-learning process are tested. Results show enhancement of actuator performance through machine learning, and the proof-of-concepts can be leveraged for continued advancement of more complex IPMC devices. Emerging challenges are also highlighted.

## Introduction

Ionic polymer metal composites (IPMC)s are electroactive-polymer soft actuators with application in biomedical devices and soft robotics. IPMCs consist of an ion-exchange membrane (also referred to as a polyelectrolyte) and electrode layers on opposing sides of the ion-exchange membrane. When hydrated and a voltage is applied across its electrodes, an IPMC deforms. Conversely, a hydrated IPMC device when deformed produces a measurable voltage and functions as a sensor^1,2^. The advantages of IPMCs include low activation voltage (<3 V), flexibility, and that they operate in aqueous environments^1^. Additionally, they are functional down to sub-micron scales^3^. Because of these advantages, IPMCs are attractive for applications such as active catheters^4^, manipulators^3,5–7^, grippers^8^, microfluidic valves and pumps^9–11^, and propulsion and sensing mechanisms in mobile robots^12–20^. However, the main challenges of working with IPMCs are the lack of novel manufacturing methods for creating complex monolithic actuator and sensor designs and limited approaches for effective actuator motion control that do not rely on complex modeling and analysis. Therefore, the main contribution of this paper is a paradigm whereby custom-shaped IPMC actuators can be manufactured as monolithic devices through 3D printing and effective motion control can be achieved through machine learning that avoids the need for modeling the complex behaviors. Furthermore, the machine learning approach is also adaptable to time-varying degradation of the material through repeated use.

The polymer matrix of the ion exchange material in IPMCs contains hydrophilic networks that form due to ionic clustering. Consequently, the hydrated ion exchange material conducts charge via the soluble counterions that neutralize the material. When a voltage is applied across the electrodes of an IPMC, charge redistributes affecting the hydrophilic regions of the polymer, causing change in volume which leads to the material deforming (*e.g*., macroscopic bending in cantilever-type actuators)^1,2^. Conversely, deforming the hydrated composite produces a voltage and thus the material acts as a sensor. IPMCs are most commonly fabricated as composites of a Nafion membrane with platinum metal electrodes, but a variety of other materials can be used to manufacture IPMCs with tuned performance characteristics or reduced cost. For example, IPMCs have been successfully manufactured from block ionomers and blends using polystyrene sulfonic acid (PSSA)^21,22^. Other membrane materials include blends of polyvinylidene fluoride (PVDF), hexafluoropropylene (HFP), polyethylene oxide (PEO), and nitrile butanide rubber (NBR)^23^. Researchers have selected membrane materials to have favorable water uptake and ion exchange capacities as well as high ion conductivity^22^. Researchers have also used additives with Nafion to improve the membrane quality. For example, these approaches include blending amphipathic organic molecules with Nafion to improve the membrane’s ion conductivity and adding hygroscopic alumina layers to reduce water loss^24^. Notably, an actuator was recently fabricated with a 300 fold improved ion conductivity and a millisecond response time through the use of a single-ion-conducting polymer as the membrane material^25^. Researchers have employed carbon nanotubes and graphene paper as the electrode layer to create highly flexible actuators that are durable and have low solvent evaporation^26^. Other conductive materials that have been used in the electrode layers include polypyrrole (PPy) or Poly(3,4-ethylenedioxythiophene) (PEDOT)^23^.

Fabricating IPMCs typically involves shaping and plating commercially-available sheets and tubular structures of Nafion, Flemion, or Aquivion material. Unfortunately, this is a laborious and unreliable means of fabricating IPMC-based actuators and devices, and thus significantly hinders the use of IPMCs in practical applications. To overcome this challenge, dispersions of ionomeric material or molten precursor to ionomeric material are used in a mold^6,27^. However, these approaches are restrictive in that new designs require new molds, which are time consuming and costly to create. In comparison to these methods, free-form-based techniques where structures are created using a layer-by-layer manufacturing process has been proposed^28,29^, where layers of Nafion are dispensed into silicone casts and the solvent in the dispersion is allowed to evaporate. This process creates both the electrode layer and the ionomeric substrate. Unfortunately, a plasticizer is required to minimize brittleness which increases drying time. The IPMCs that have been created had lower blocking force compared to traditionally-made IPMCs^28^. Alternatively, it has been shown that fused deposition modeling (3D printing) can be used to deposit a precursor to an ionomeric material as a monolithic body which can subsequently be functionalized and plated, creating IPMCs of any desired shape^20,30^. However, with increasingly complex actuator designs, IPMCs become increasingly difficult to control for practical applications.

There are a number of challenges to controlling IPMCs. Specifically, two identical IPMC-based actuators, fabricated through the same process will exhibit appreciably different behaviors^31^. Also, the behavior of a single IPMC-based actuator will vary over time^31^ due to solvent evaporation, aging, and degradation from repeated use. IPMCs also exhibit nonlinearity and other complex dynamic behavior such as back-relaxation and higher order resonances^32^. Additionally, sophisticated, custom-shaped, multi-input-multi-output (MIMO), IPMC-based systems (as may be fabricated through 3D printing) will exhibit coupled nonlinear behavior that may be difficult to model and control. To address these challenges, prior works have developed sophisticated control-oriented dynamics models and advanced feedback-control methods to compensate for time-varying and complex dynamic behavior. However, existing models are limited in their applicability to custom-shaped IPMCs. Moreover, unresolved challenges to integrating IPMCs with sensors inhibits the use of feedback control^33,34^. A possible solution to these challenges is the use of feedforward learning-based control methods such as Bayesian optimization. These methods are especially applicable to devices that operate repetitively, where the processes of iteration can be exploited to evaluate and adjust control inputs that optimizes a relevant performance metric. Recent work on Bayesian optimization has shown it to succeed in spite of complex, idiosyncratic, and time-varying behavior^35,36^. These features, together with the ability of Bayesian optimization to incorporate prior knowledge to speed up convergence, make it an attractive candidate for use on more complex 3D-printed IPMC actuators and devices.

Currently, there are other closely-related soft actuators with similar challenges to IPMCs. For instance, hydrogels are a stimuli responsive material that have the potential for use in a variety of biomedical and robotic applications^37^. When these gels are realized as a polyelectrolytes and immersed in an electrolytic environment, they can be controlled by electric fields^38^. The electric field causes the counter-ions to migrate, leading to a concentration gradient that causes bending of the actuator^38^. In order to fabricate custom-shaped hydrogel actuators, a variety of 3D-printing techniques have been developed including extrusion-based, steriolithographic, and jetting-based methods^37^. Control of hydrogels is generally through thermal or hygroscopic stimuli, while polyelectrolyte gels are also stimulated by electric fields^38^. Lacking electrodes, polyelectrolyte gels are not individually controllable and must be immersed in an electrolytic environment in order to respond to electric stimuli. The response time of hydrogels is also significantly slower than other ionic electroactive polymers^37^. Another closely-related material to IPMCs are conjugated polymer actuators. Conjugated polymers, such as polypyrrole (PPy) and polyaniline (PANI), which have alternating single and double bonds, are organic semiconductors. When a sufficiently-large voltage is applied to a conjugated polymer, electrons are removed. If the material is in contact with an electrolyte, the material will then change size in proportion to its change in oxidation level^39^. Conjugated polymer actuators are generally layered bending actuators like IPMCs, involving a composite of a conjugated polymer with at least one noble metal electrode layer^40^. Addition of polyelectrolyte gels or electrolyte infused membranes to such composites was developed to allow the actuator to operate outside of an electrolytic environment^41^. Well established micro-machining techniques have been developed for fabricating conjugated polymer actuators^40^, but not a 3D printing process. 3D printing has been developed for some conjugated polymers, but not for the composite necessary for fabricating an actuator^42^. Like IPMCs, the behavior of polyelectrolyte gels and conjugated polymer actuators is complex and time varying, and there are significant challenges to integrating sensors^43–46^. The machine learning approach proposed here is equally relevant to these materials to help improve performance.

To address the challenges to manufacturing and control of IPMCs and related materials, this paper considers 3D printing for manufacturing and machine learning to control example IPMC actuator components, which together can be assembled to create a modular reconfigurable soft crawling robot platform to demonstrate feasibility. The development of the platform is presented to illustrate the proof-of-concept of 3D-printing of IPMC actuators and application of machine learning for effective motion control. Since the use of Bayesian optimization on IPMCs is new, this paper tests two hypotheses: (1) that Bayesian optimization will lead to convergence in fewer trials than a finite-difference policy gradient method making it more suitable for controlling IPMCs; and (2) that prior knowledge from a dynamics model (especially a known achievable target value) will lead to convergence in fewer number of trials than simply optimizing from a uniform prior distribution.

To investigate these hypotheses, first, the linear dynamics of the 3D-printed soft crawling platform are modeled and then validated through simulation and in physical experiments. The dynamics model of the robot is then used to contrast the performance of Bayesian optimization and finite-difference policy gradient method in simulation, where convergence is achieved in fewer trials using Bayesian optimization^35,47,48^. Since these simulations show the superiority of Bayesian optimization, the process is then applied to the real IPMC-based system. Experiments are presented to compare the performance of Bayesian optimization from a uniform prior with the performance of Bayesian optimization using a prior distribution obtained from optimizing in simulation on the dynamics model.

The contribution of this work is 3D printing of IPMCs and machine-learning control of the 3D-printed IPMCs and related actuators. The 3D-printing process, design and modeling of the actuator components, and Bayesian optimization as a learning-based control method are discussed in detail. Additionally, this paper compares the effectiveness of a finite-difference policy gradient method and Bayesian optimization as control methods for IPMCs. The results of the comparison highlight the advantage of Bayesian optimization’s ability to encode prior knowledge in the form of known reachable target values and simulation results from a linear dynamics model.

## Methods

### 3D Printing of IPMCs

**Figure 1:**
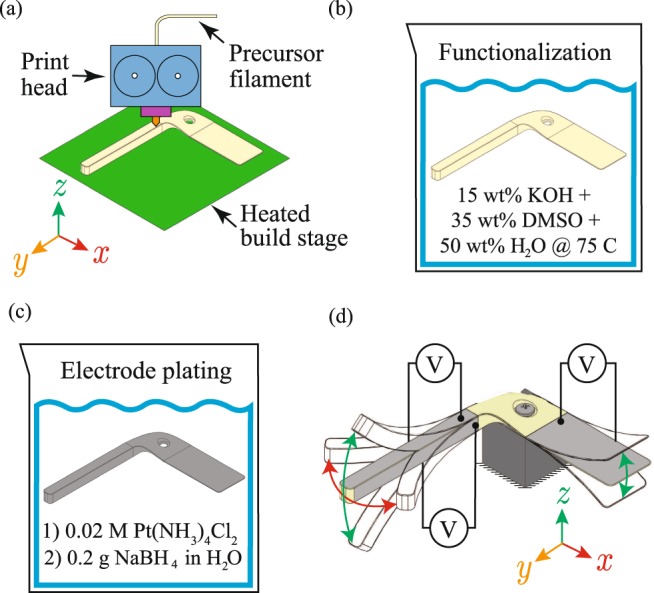
Fused-filament 3D printing process for IPMCs: (**a**) printing of soft structure using precursor of ionomeric material, (**b**) functionalization of printed precursor structure, (**c**) plating process, and (**d**) segmenting and wiring of electrodes for multi-DOF actuation and/or sensing.

The 3D-printing process used to fabricate custom-shaped IPMCs involves four major steps as illustrated in Fig. 1. First, the fused-filament fabrication process requires manufacturing filament using Nafion’s (or Aquivion’s^49^) sulfonyl fluoride precursor. Next, a custom-designed 3D printer is required to process the precursor material for 3D printing. Since the precursor is not electroactive immediately after being 3D printed, an *in situ* functionalization process is then employed to convert the precursor material to fully ionomeric Nafion. Subsequently, the ion-exchange properties of the ionomeric material is leveraged in an electroless plating process to create platinum electrodes on the ionomeric material’s surface.

#### Filament extrusion and 3d printing of ionomeric precursor

Since commercially-available ionomeric materials, such as Nafion (typically, in the sulfonic acid form −SO_3_^−^H^+^), are not melt processable^50,51^, the sulfonyl fluoride precursor of Nafion (−SO_2_F), sometimes referred to as XR resin, is employed. Nafion precursor filament is created using a filament extruder^52^. For example, XR resin is fed into a hopper system, where a drive mechanism, such as an auger or a piston, advances the precursor material for extrusion through a nozzle to form the filament. Cooling and a drawing mechanism may also be used to maintain dimensional consistency. Typical extrusion parameters include extrusion temperature range between 220 to 300 °C, and extrusion and drawing rates between 10 to 125 mm/s, depending on desired filament diameters.

**Figure 2:**
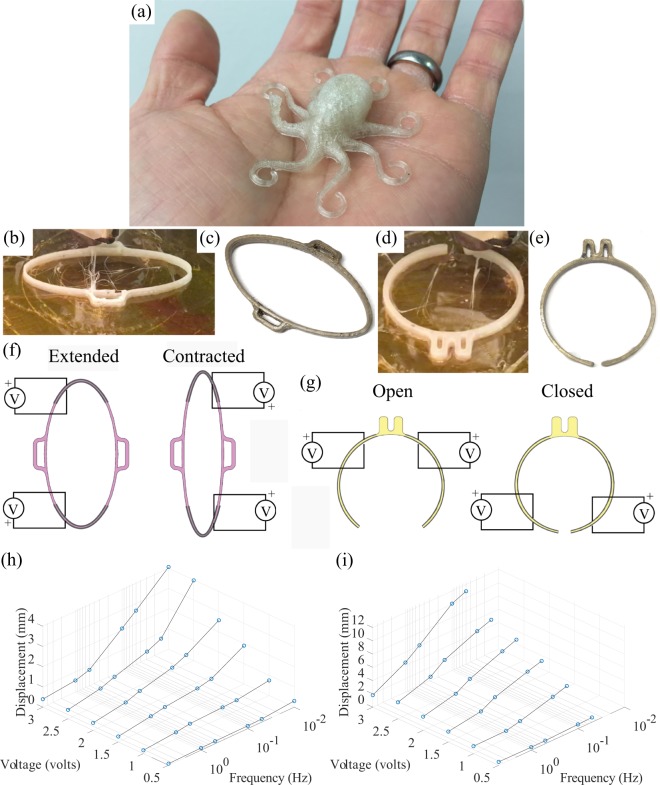
Example 3D-printed Nafion precursor and functionalized and plated devices: (**a**) monolithic Nafion precursor octopus-shaped device prior to functionalization and electrode application; (**b**,**c**) linear actuator component for modular robotic device, printed, functionalized, and plated examples; (**d**,**e**) gripper mechanism for modular robotic device, printed, functionalized, and plated examples; (**f**) 3D-printed linear actuator extension and contraction as a function of applied voltage; (**g**) 3D-printed gripper actuator opening and closing as a function of applied voltage; (**h**) peak displacement response of linear actuator for a range of amplitudes and frequencies of the input signal, (i) peak displacement response of gripper for a range of amplitudes and frequencies of the input signal.

There are several challenges to 3D printing the Nafion precursor material including Nafion’s high glass-transition temperature, poor adhesion, material compressibility, and the tendency for the material to buckle in the drive gears of conventional 3D printers. These issues make it challenging to control the rate at which the material is extruded from the nozzle. Commercial 3D print heads cannot effectively handle the Nafion precursor material without modifications, thus a custom-designed system is often required. Such custom print heads can be designed and integrated into commercial motion control platforms. Additionally, the build stage must be able to heat to approximately 200 °C to enhance adhesion characteristics during printing. A constrained filament path is often needed to prevent filament buckling. A thermal barrier, which confines the transition and melt zones to the heater block and heated nozzle, prevents the filament from progressively softening as it approaches the heater block. The components of the heater block can reach temperatures exceeding 300 °C to melt the filament material. The melted filament collects in the nozzle and is extruded out onto a high temperature print bed to ensure that the printed material adheres^52^. More recently, it was determined that commercially-available print heads designed for soft polymer materials can be adapted to print Nafion precursor by incorporating a heater capable of reaching approximately 300 °C. Figure 2(a,b,d) show examples of 3D-printed Nafion precursor devices.

#### Functionalization process

After printing, the component has to be made electroactive through a functionalization process. This involves converting the non-ionomeric Nafion precursor into fully ionomeric Nafion, which has sulfonate terminal groups that give Nafion its characteristic ion conducting ability. This process is done by base hydrolysis using a solution of 15 wt% KOH, 35 wt% DMSO, and 50 wt% deionized water at 75 °C as prescribed by the manufacturer^53^. In this process, Florine is exchanged nucleophilicly with a hydroxyl group, and the hydrogen ion is then exchanged electrophilicly for a potassium ion. A hydration sphere then forms around the potassium ion which swells the material and permits the ingress of the functionalizing solution. At 75 °C, the process proceeds at a rate of approximately 1.3 *μ*m per minute^54^. The printed components are left in the functionalization solution for additional time (approximately 4 hours) after the estimated completion of the hydrolysis process, to facilitate the development of ionic clusters^54^. The material is then rinsed in two successive baths of deionized water at 75 °C for 30 minutes each time. To confirm complete hydrolysis (*i.e*., functionalization), the material can be stained using methylene blue, which only dyes the “activated” material^54^.

#### Electrode plating process

The ion-exchange properties of the now ionomeric material can be leveraged in an electroless plating process. Following functionalization, the material is converted to its acid form using 20% nitric acid (sulfuric acid can also be used). This exchanges the potassium ions neutralizing the material with hydrogen ions and is done as an intermediate step whenever attempting to change the neutralizing ionic species. The material is then rinsed again in deionized water, following which it is immersed in a 0.2 molar solution of tetraamineplatinum (II) chloridemonohydrate ([Pt(NH_3_)_4_] · Cl_2_ × H_2_O, Alfa Aesar) for a period of 2 to 4 hours. This deposits platinum ions in the surface layer of the Nafion. The platinum ions are then converted to elemental platinum by placing them in deionized water and repeatedly introducing a reducing agent such as sodium borohydride (NaBH_4_, Aldrich) into the solution over the course of 3 hours. Repetition of this process two or three times will significantly improve the responsiveness of the resulting IPMC. Subsequently, either electrical or electroless plating methods can be used to further develop these electrodes and decrease their surface resistance. The results of plating are shown in Fig. 2(c) and (e) for the printed robot body and leg components, respectively.

### Fabrication and characterization of 3d-printed IPMC actuators

**Figure 3:**
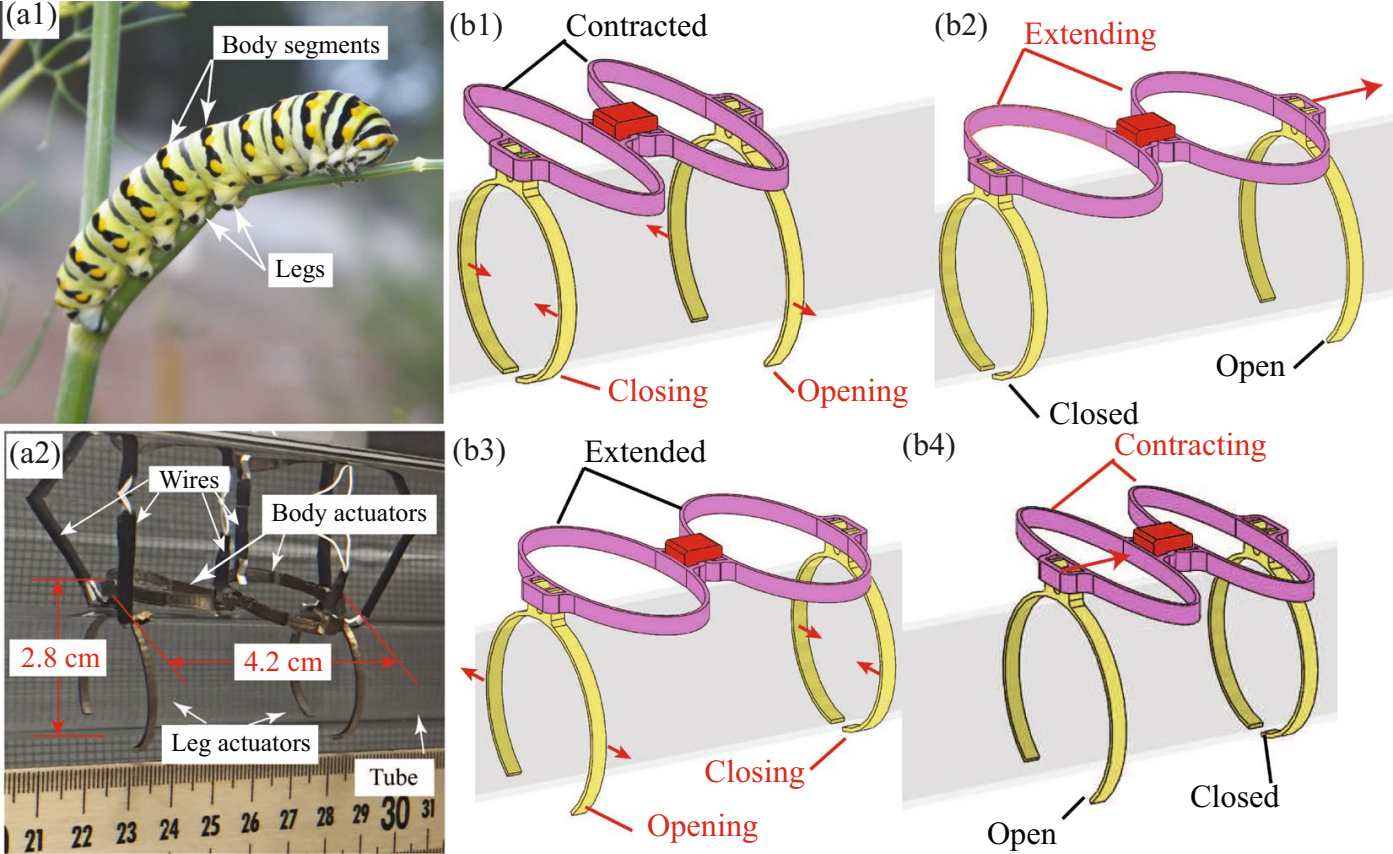
Example 3D-printed IPMC soft crawling robot inspired by a caterpillar: (**a1**) biological caterpillar showing body and leg segments that were modeled in the design of the crawling robot; (**a2**) assembled robot with two legs and two body sections, with electrical connections; (**b1**) back leg actuator closing and front leg actuator opening while body is contracted; (**b2**) extension of body actuators while back leg actuator is closed and front leg actuator is open propelling the robot forward; (**b3**) back leg actuator opening and front leg actuator closing while body is extended; (**b4**) contraction of body actuators while back leg actuator is open and front leg actuator is closed drawing up the back of the robot.

The IPMC 3D-printing process was used to fabricate a variety of custom-shaped IPMC actuators as shown in Fig. 2. These include an octopus printed in Nafion precursor material, as shown in Fig. 2(a), as well as an IPMC-based linear actuator, as shown in Fig. 2(b,c), and an IPMC-based gripper, as shown in Fig. 2(d,e). The linear actuator and gripper were first 3D-printed using the fused-filament technique as described above. This consisted of first printing the desired geometry using the precursor filament material, then applying chemical activation, followed by plating and conditioning the components, and finally segmenting the plated electrodes on the surfaces to create independent electrode pairs. As shown in Fig. 2(b,d), the printed product has a yellowish discoloration. The material is soft and pliable and has a Teflon-like texture. After the subsequent chemical processes, the resulting ionomeric material beneath the plated surface is clear and though still distensible, is stiffer than the original printed product. As can be seen in Fig. 2(c,e), the plated linear actuator and gripper have a dull gray coloration after the two step plating process. This indicates a high conductivity across the plated surface. The linear actuator is elliptically-shaped with its interior and exterior electrodes segmented into four distinct regions, as shown in Fig. 2(f). The linear actuator extends with an applied voltage such that the exterior is the anode and the interior is the cathode. The linear actuator contracts with applied voltage such that the exterior is the cathode and the interior is the anode. The linear actuator has pockets for coupling with other devices such as the 3D-printed gripper. As shown in Fig. 2(g), the gripper is nominally a circular structure designed to fit around a tube. The gripper has two independent electrode pairs on the inside and outside of the individual arms, as shown in Fig. 2(h). When a positive voltage is applied such that the exterior electrodes are the anode side and the interior electrodes are the cathode side, the gripper opens, as shown in Fig. 3(d). When the voltage is applied to these electrode pairs in opposite polarity, the gripper closes, as shown in Fig. 2(h). Each gripper has a protrusion that extends off of it which engages in an interference fit with other devices, such as the 3D-printed linear actuators.

As shown in Fig. 2(h,i), periodic voltage signals were applied to the linear actuator and gripper over a range of amplitudes and frequencies to characterize the actuation performance. The linear actuator reaches its maximum displacement in response to 3 volt signals applied at 0.01 Hz, as can be seen in Fig. 2(h). The gripper reaches its maximum displacement in response to 3 volt signals applied at 0.05 Hz, as can be seen in Fig. 2(i). The actuation performance was deemed appropriate for developing a soft robotic testbed to demonstrate feasibility, as described below.

### Example modular reconfigurable soft crawling platform

There has been prior work on the additive manufacturing of soft actuators for soft robotic platforms, but the approaches required assembly with actuators and sensors after fabricating the soft robot body^55,56^. Notably, researchers have very successfully constructed soft robots based on pneumatic and fluid elastomeric actuators^57–62^. Recently, multi-material additive manufacturing methods have even been successfully employed to create soft robotic devices^63,64^. These devices utilize pressure gradients provided by hosing, internal chemical reactions or electric motors for actuation. Consequently, the scale of the robot is limited by the size of the mechanisms required for actuation. Also, recent research has developed “4D printing” and origami-based robots, that are able to reconfigure into a functional shape after being fabricated (often as a flat sheet). These are very promising, but shape change is generally accomplished *via* heating or other nonelectric stimuli^65–68^. In general, the development of functional soft electroactive polymer based robotic platforms is limited. Thus, an important complimentary approach to these other techniques is the proposed 3D-printing process to create actuators for realizing soft robotic systems. As an example application to demonstrate proof-of-concept, the linear actuators and grippers shown in Fig. 2(b–e) are used to create a modular reconfigurable soft crawling robot. The 3D-printed soft IPMC robot is inspired by a caterpillar or worm-like organism, as shown in Fig. 3(a1). The robot platform consists of modular grippers that act as its legs and linear actuators that act as its body segments. The robot is designed to “grip” onto a cylindrical tube and “inch” its way along the tube, as shown in Fig. 3(b1–b4). The modular components can be assembled into various configurations to create a robot of varying lengths. A leg-body segment of a crawling robot is assembled from a gripper and linear actuator. These segments can be chained together to create longer sections of the robot. The robot can be assembled into multiple configurations to test which results in faster locomotion and how the different configurations effect gait performance. An example of the locomotion of a simple leg-body-body-leg robot is illustrated in Fig. 3(b1–b4). In this configuration, the nominal gait begins with the linear actuators contracted while the rear gripper closes and front gripper opens, as shown in Fig. 3(b1). Then, the linear actuators extends propelling the front end of the robot forward, as shown in Fig. 3(b2). Next, the front gripper closes and the rear gripper opens, as shown in Fig. 3(b3). Finally, the linear actuators contract, as shown in Fig. 3(b4), bring the rear end of the robot towards the stationary front end. The process is repeated enabling the robot to move forward.

### Dynamics modeling and parameter estimation

The actuation behavior of the 3D-printed IPMC actuators and gripper mechanisms described above are modeled and the model is used to predict the performance of the overall IPMC-based robotic testbed. Additionally, the model is exploited by the machine learning algorithm for gait optimization.

#### Electromechanical model

**Figure 4:**
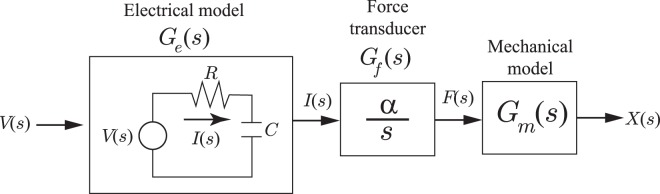
An IPMC model: The applied voltage *V*(*s*) is the input to an equivalent electrical circuit model, where current *I*(*s*) is the output. A force transducer model (integral term) relates current *I*(*s*) to force *F*(*s*). Finally, the force is applied to a mechanical model where the output is the displacement of the IPMC mechanism.

The electromechanical dynamics of an IPMC actuator consists of an electrical model, force transducer, and mechanical model as illustrated in Fig. 4. The electrical model is an equivalent circuit that relates the applied voltage *V*(*s*) (where ‘*s*’ is the Laplace variable) to the current *I*(*s*). The current *I*(*s*) then becomes the input to the force transducer *G*_*f*_(*s*), modeled by an integral term, where the output is the mechanical force *F*(*s*). Finally, the force drives a lumped-parameter model of the structural dynamics of the actuator with output displacement *X*(*s*).

The transfer function that relates the applied voltage *V*(*s*) to the output current *I*(*s*) is given by

1 $${G}_{e}(s)=\frac{I(s)}{V(s)}=\frac{\frac{1}{R}s}{s+\frac{1}{RC}}.$$

In this model, *R* is the effective electrical resistance and *C* is the effective capacitance of the actuator.

Assuming that the effective IPMC force *F*(*s*) is linearly related to the density of the transferred charges^3^, then

2 $${G}_{f}(s)=\frac{F(s)}{I(s)}=\frac{\alpha }{s},$$

where *α* is a constant. It is noted that this model is only valid over operating frequencies where back-relaxation effect, which is characteristic of IPMCs, is not significant^69^.

**Figure 5:**
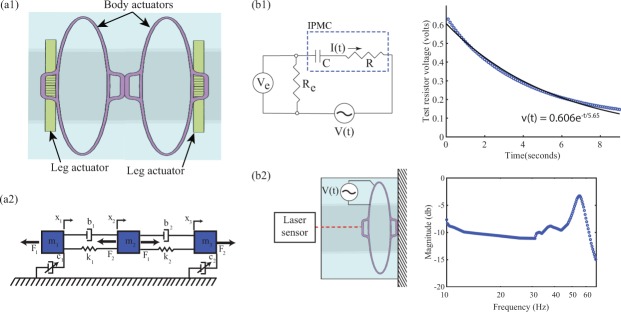
Modeling and parameter estimation of crawling robot: (**a1**) two body actuators and two leg actuators configured as a crawling robot; (**a2**) model of crawling robot as a chain of lumped masses, springs and dampers where *m*_1_, *m*_2_, and *m*_3_ are effective masses, *k*_1_ and *k*_2_ are springs, *b*_1_ and *b*_2_ are dampers, and *c*_1_ and *c*_2_ are damping elements that approximate the viscous friction between the legs and the tube; (**b1**) equivalent RC-circuit model for the IPMC actuator and the measured step response of the body-segment behavior compared to the model *V*(***t***) = 0.6*e*^*t*/5.65^ (R-squared value of 0.9911), where *R*_*e*_ = 220Ω. (**b2**) schematic of the experiment for characterizing mechanical model parameters and the measured experimental frequency response of the actuator.

The robot’s structural dynamics, *G*_*m*_(*s*), is modeled by inertial, elastic, and damping elements, as shown in Fig. 5(a2). The variable damping elements, *c*_1_, …, *c*_*n*_, shown in Fig. 5(a2), model the frictional contact behavior between the legs and the tube. Balancing forces on the masses in Fig. 5(b2) yields the following relationships:

3 $${m}_{1}{\ddot{x}}_{1}={b}_{1}({\dot{x}}_{2}-{\dot{x}}_{1})-{c}_{1}{\dot{x}}_{1}+{k}_{1}({x}_{2}-{x}_{1})-{F}_{1},$$

4 $$\begin{array}{rcl}{m}_{i}{\ddot{x}}_{i} & = & {b}_{i-1}({\dot{x}}_{i-1}-{\dot{x}}_{i})+{b}_{i}({\dot{x}}_{i+1}-{\dot{x}}_{i})-{c}_{i}{\dot{x}}_{i}+{k}_{i-1}({x}_{i-1}-{x}_{i})\\  &  & +{k}_{i}({x}_{i+1}-{x}_{i})+{F}_{i-1}-{F}_{i},\end{array}$$

5 $${m}_{n}{\ddot{x}}_{n}={b}_{n-1}({\dot{x}}_{n}-{\dot{x}}_{n-1})-{c}_{n}{\dot{x}}_{n}+{k}_{n-1}({x}_{n}-{x}_{n-1})+{F}_{n-1},$$

where *x*_1_, …, *x*_*n*_ are the displacements of the effective masses *m*_1_, …, *m*_*n*_, respectively; *b*_1_, …, *b*_*n*−1_ and *k*_1_, …, *k*_*n*−1_ are the constants for the damping and elastic elements, respectively; and *F*_1_, …, *F*_*n*−1_ are the forces applied to each inertial element.

The equations of motion can be written in the following matrix form:

6 $$[\begin{array}{c}\dot{\overrightarrow{p}}\\ \dot{\overrightarrow{q}}\end{array}]=[\begin{array}{ll}{\tilde{A}}_{11} & {\tilde{A}}_{12}\\ {\tilde{A}}_{21} & \tilde{0}\end{array}]\,[\begin{array}{l}\overrightarrow{p}\\ \overrightarrow{q}\end{array}]+\,[\begin{array}{l}{\tilde{B}}_{1}\\ \tilde{0}\end{array}]\overrightarrow{F},$$

where $\overrightarrow{p}$, $\overrightarrow{q}$, and $\overrightarrow{F}$ are vectors of the momentums, displacements, and forces, respectively. Matrices ${\tilde{A}}_{11}$, ${\tilde{A}}_{12}$ and ${\tilde{A}}_{21}$ have dimension *n* × *n* and ${\tilde{B}}_{1}$ has dimension *n* × (*n* − 1). The vectors $\overrightarrow{p}$ and $\overrightarrow{q}$ have dimension *n* × 1 and $\overrightarrow{F}$ is dimension (*n* − 1) × 1. For an example robot with two bodies and three legs, ${\tilde{A}}_{11}$, ${\tilde{A}}_{12}$, ${\tilde{A}}_{21}$ and ${\tilde{B}}_{1}$ are given by

$${\tilde{A}}_{11}=[\begin{array}{ccc}\frac{-{c}_{1}-{b}_{1}}{{m}_{1}} & \frac{{b}_{1}}{{m}_{2}} & 0\\ \frac{{b}_{1}}{{m}_{1}} & \frac{-{c}_{2}-{b}_{1}-{b}_{2}}{{m}_{2}} & \frac{{b}_{2}}{{m}_{3}}\\ 0 & \frac{{b}_{2}}{{m}_{2}} & \frac{-{c}_{3}-{b}_{2}}{{m}_{3}}\end{array}],$$

$${\tilde{A}}_{12}=[\begin{array}{ccc}-{k}_{1} & {k}_{1} & 0\\ {k}_{1} & -{k}_{1}-{k}_{2} & {k}_{2}\\ 0 & {k}_{2} & -{k}_{2}\end{array}],$$

$${\tilde{A}}_{21}=[\begin{array}{lll}\frac{1}{{m}_{1}} & 0 & 0\\ 0 & \frac{1}{{m}_{2}} & 0\\ 0 & 0 & \frac{1}{{m}_{3}}\end{array}],{\rm{and}}\,{\tilde{B}}_{1}=[\begin{array}{ll}-1 & 0\\ 1 & -1\\ 0 & 1\end{array}].$$

The output of the model given by Eq. (6) can be compared to experimental results to validate the robot’s gait performance. Furthermore, the model can be used to help design a controller to enhance the robot’s gait.

#### Identifying the model parameters

From Fig. 5(b1), the transfer function for circuit model relating the applied voltage *V*(*s*) to the voltage *V*_*e*_(*s*) across the resistor *R*_*e*_ is given by

7 $$\frac{{V}_{e}(s)}{V(s)}=(\frac{{R}_{e}}{R+{R}_{e}})(\frac{s}{s+\frac{1}{\tau }}),$$

where *τ* = (*R* + *R*_*e*_)*C*. Then for a step input, the time response is

8 $${v}_{e}(t)=(\frac{{R}_{e}}{R+{R}_{e}}){e}^{-\frac{t}{\tau }}.$$

To find the model parameters, a step input is applied to the IPMC and the measured voltage across a series resister with the IPMC is used to determine the parameters of the electrical model through curve-fitting. Table 1 lists the experimentally-determined parameters of the electrical model, where “n/m” denotes “not measured”.

**Table 1:** Values for parameters of the electromechanical model, where “N/M” denotes not measured.

	R (Ohms)	C (mF)	*τ* (sec)	*ω*_*c*_ (rad-sec^−1^)	m_1_ (g)	m_2_ (g)	b (Dyn-s-cm^−1^)	c (Dyn-s-cm^−1^)	k (N-cm^−1^)
Body-actuator one	140	16	2.2	0.45	1.4	1.0	59	56	1.30
Body-actuator two	260	24	6.2	0.16	1.2	0.7	63	57	0.65
Leg-actuator one	100	55	5.6	0.17	N/M	N/M	N/M	N/M	N/M
Leg-actuator two	170	56	9.5	0.11	N/M	N/M	N/M	N/M	N/M
Leg-actuator three	210	52	10.9	0.09	N/M	N/M	N/M	N/M	N/M

For the mechanical model, the effective spring constant is determined by the ratio between an applied known load and the measured actuator deflection. The equivalent mass and damping constant are determined by analyzing the measured frequency response (obtained with an HP 35665 A digital signal analyzer) of the IPMC actuator, as shown in Fig. 5(b2). The boundary conditions for these experiments are shown in Fig. 5(b2), where one side of a body or joined body and leg are fixed and the displacement of the free end is measured by a laser displacement sensor (Keyence LK-2001). For body sections, experiments were conducted both with an attached gripper (leg) and without a gripper. More details about the experimental parameter identification process can be found in prior work^20^.

### Machine learning control via bayesian optimization

Modeling, control, and higher level planning of IPMC-based actuators is complicated by both the flexibility of the material as well as the material’s complex electrochemical behavior. The flexibility of the material can potentially be addressed in manners similar to that of other soft and elastomeric devices^70^. As with pneumatic or fluid elastomer actuators, curvature is controlled in IPMC-based systems. Kinematic models can thus be developed by modeling IPMC-based systems with piece-wise functions that describe segments’ deformations from their original undeflected states^3,71^. Further, the mechanical behavior of IPMCs can be modeled similar to that of other polymers or elastomers^72^. But the response of IPMCs to electrical inputs suffers from non-repeatability, nonlinear effects, and there are still significant challenges to integrating sensors on IPMC actuators to measure state. These difficulties make it difficult to implement conventional feedback controllers^32,33^. Machine learning control (which encompasses a wide variety of techniques) is an attractive solution for control of IPMC-based devices because, like conventional adaptive control, the approach can adjust control policy parameters to address non-repeatability and complex non-linear effects. However, the fundamental difference between conventional adaptive control and machine learning control is that conventional adaptive control theoretically employs continuous-time feedback signals, whereas machine learning control is an iterative process, where improvements are done from one operating cycle to the next. Consequently, the requirements for sensor feedback can be minimal for some machine learning control techniques. For instance, use of direct policy search methods in highly repetitious tasks (like gait optimization for a walking robot or optimization of the stroke of a diaphragm pump) are excellent examples of machine learning being employed with minimal sensor feedback. In these cases, a gross performance metric is only sampled once at the end of an iteration (such as a period of attempted walking or a period of attempted pumping). This is particularly relevant to soft actuators because in practical applications it may often be possible to take periodic measurements of cumulative performance (like distance traveled or volume of fluid pumped) but not to continuously monitor a system output (like actuator displacement). This use of reinforcement learning reflects a larger trend in which control methods are being developed to suite the compromised accuracy, repeatability, and internal sensing capabilities of low-cost robots and devices^73^.

Direct policy search methods belong to the broader family of reinforcement learning which is extensively used in soft robotics. Reinforcement learning iteratively evaluates the reward associated with states, policies and/or actions in an attempt to optimize a control policy. Broadly speaking, there are three kinds of reinforcement learning methods: model-based methods, value-based model-free methods, and policy-based model-free methods (or direct policy search methods). Model-based methods attempt to learn a model of the system (generally in the form of a Markov decision process) and then solve for the optimal control policy based on cumulative reward^73,74^. Most recently, model-based methods employing neural networks as models have been deployed on a variety of platforms including a tensegrity-based mobile robot and a pneumatic manipulator^75,76^. Value-based model-free methods attempt to learn a function of the total possible cumulative reward, but do not employ a state transition distribution. Most recently, the temporal difference (TD) algorithm has been employed to control the position and stiffness of a fluid actuated soft robotic arm^77,78^ and Q learning has been employed to learn an effective control policy for the dielectric elastomer actuators employed in an artificial cuttlefish robot^79^. In contrast to these methods, policy-based model-free methods do not employ either a model or a cumulative value function, but rather directly optimize control parameters in the policy space^74^. Direct policy search methods include policy gradient methods^47^ and genetic algorithms^80^. The general advantages of direct policy search methods are that they are simpler and often are more computationally efficient.

Recently, Bayesian optimization has been successfully employed as a direct policy search method to learn a walking gait for a biped robot^35,81^. In comparison to gradient-based methods and methods employing genetic algorithms, Bayesian optimization converges in significantly fewer iterations and is theoretically guaranteed to find the global optimum^82^. Bayesian optimization assumes the objective function can be modeled as a Gaussian process (GP) and utilizes all previous evaluation points to select future ones. Therefore, it sacrifices computational efficiency to make better use of available data^48^. Previous works have emphasized Bayesian optimization’s ability to function as a black box optimizer without requiring expert knowledge (such as in the form of a dynamics model)^35,74^. However, another notable feature of Bayesian optimization is precisely its ability to incorporate incomplete or imperfect expert knowledge to speed up convergence to the true global optimum. This can be done by encoding known reachable performance targets. It can also be done by incorporating a prior distribution based on simulation data that, while not capturing every aspect of a system dynamics, is known to be generally accurate^74^. In previous uses of Bayesian optimization since optimization was conducted without the benefit of a model to generate simulated results, the selection of the initial evaluation points were effectively random^35^. In one of the methods employed here, a dynamics model of the robot is exploited and Bayesian optimization is conducted first in simulation on the model. The Gaussian process trained in simulation is then used as the prior for Bayesian optimization on the real robot.

In deploying Bayesian optimization on the crawling robot, the goal is to determine the input amplitudes and relative phases of the inputs that are applied to the leg and body section such that the robot achieves robust locomotion. To do so, an effective crawling gait is learned using Bayesian optimization. In this approach, a Gaussian process (GP) models robot speed, *f*(*θ*), over a domain of gait parameters, *θ* (the phase and amplitudes of inputs into the robot actuators). The GP is trained from the set of past evaluation points of the objective function, that constitutes the training data, ${\mathbb{T}}$. The GP is then mapped to a surface *via* an acquisition function. A new evaluation point is selected by optimizing over the resultant surface^35^. This technique is employed first on the robot in simulation and then on the real robot experimentally using the GP from optimizing in simulation as the prior distribution. The general Bayesian optimization algorithm is given in Alg. 1.

**Algorithm 1:**
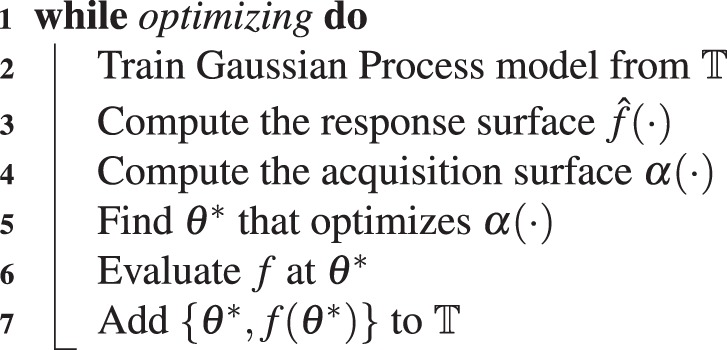
Bayesian optimization algorithm.

A Gaussian process is used in Bayesian optimization to define what is termed the “virtual objective function”, $\hat{f}(\theta )$. The Gaussian process models the relation between parameters, *θ*, and the experimental performance results from testing those parameters, *f*(*θ*), as a distribution over functions^35^. The function $\hat{f}(\theta )$ is defined by a mean function *m*(*θ*) and a covariance function *k*(*θ*):

$$\hat{f}(\theta )\sim GP(m(\theta ),k(\theta ,\theta ^{\prime} )).$$

The virtual objective function is used to predict probable performance results f_*_ at selected parameter values Θ_*_. These future predictions are modeled as jointly Gaussian with prior data, {Θ,**f**}. This is expressed as follows:

$$[\begin{array}{l}{\bf{f}}\\ {{\bf{f}}}_{\ast }\end{array}]\sim N(\begin{array}{ll}\begin{array}{l}{\bf{m}}(\Theta )\\ {\bf{m}}({\Theta }_{\ast })\end{array}, & [\begin{array}{ll}K(\Theta ,\Theta ) & K(\Theta ,{\Theta }_{\ast })\\ K({\Theta }_{\ast },\Theta ) & K({\Theta }_{\ast },{\Theta }_{\ast })\end{array}]\end{array}),$$

where **m** is a vector of mean values and *K* is the covariance matrix^83^. It follows that the posterior probability distribution of f_*_ is given by the equation,

9 $$\begin{array}{c}{{\bf{f}}}_{\ast }|{\Theta }_{\ast },\Theta ,{\bf{f}}\sim N({\bf{m}}({\Theta }_{\ast })+K({\Theta }_{\ast },\Theta )K{(\Theta ,\Theta )}^{-1}({\bf{f}}-{\bf{m}}(\Theta )),\\ K({\Theta }_{\ast },{\Theta }_{\ast })-K({\Theta }_{\ast },\Theta )K{(\Theta ,\Theta )}^{-1}K(\Theta ,\Theta \ast )).\end{array}$$

In the case of a zero prior mean, Eq. (9) becomes

10 $$\begin{array}{c}{{\bf{f}}}_{\ast }|{\Theta }_{\ast },\Theta ,{\bf{f}}\sim N(K({\Theta }_{\ast },\Theta )K{(\Theta ,\Theta )}^{-1}{\bf{f}},\\ K({\Theta }_{\ast },{\Theta }_{\ast })-K({\Theta }_{\ast },\Theta )K{(\Theta ,\Theta )}^{-1}K(\Theta ,\Theta \ast )).\end{array}$$

If a model of the objective function exists, Eq. (10) can still be used to predict the error between future predictions and predictions of the existing model. In that case error is defined as follows: *e*(*θ*) = *f*(*θ*) − *f*_*e*_(*θ*), where *e*(*θ*) is the error and *f*_*e*_(*θ*) is the prediction of the existing model at *θ*. Equation (10) then becomes

$$\begin{array}{c}{{\bf{e}}}_{\ast }|{\Theta }_{\ast },\Theta ,{\bf{e}}\sim N(K({\Theta }_{\ast },\Theta )K\,{(\Theta ,\Theta )}^{-1}{\bf{e}},\\ K({\Theta }_{\ast },{\Theta }_{\ast })-K({\Theta }_{\ast },\Theta )K{(\Theta ,\Theta )}^{-1}K(\Theta ,\Theta \ast )).\end{array}$$

Subsequently, *f*(θ^*^) can then be determined from *f*(θ^*^) = *e*(θ^*^) + *f*_*e*_(θ^*^), which is the summation of two normally distributed random variables. Use of the error requires modification to the Bayesian optimization, however. The Gaussian process is now trained on the error, but optimization is still conducted with respect to $\hat{f}(\theta )$. Therefore, computing the response surface involves combing the existing model, ${\hat{f}}_{e}(\theta )$, and the predicted error, $\hat{e}(\theta )$. Moreover, the actual error at an evaluation point must be computed from the difference between the actual performance and the prediction of the existing model. This modified Bayesian optimization algorithm is shown in Alg. 2.

Here, the covariance function used is the squared exponential kernel, which is defined as

11 $$k({\theta }_{{\bf{p}}},{\theta }_{{\bf{q}}})={\sigma }_{f}^{2}\exp (-\frac{1}{2}{({\theta }_{{\bf{p}}}-{\theta }_{{\bf{q}}})}^{T}\lambda ({\theta }_{{\bf{p}}}-{\theta }_{{\bf{q}}}))+{\sigma }_{w}^{2}{\delta }_{pq},$$

where *σ*_*f*_^2^ is the functional variance, *λ* is a diagonal matrix of characteristic lengths, *σ*_*w*_^2^ is the noise variance, and *δ*_*pq*_ is the Kronecker delta. The parameters *σ*_*f*_^2^, *λ*, and *σ*_*w*_^2^ are set by way of automatic relevance determination. The acquisition function employed to map the response surface to the acquisition surface is the probability of improvement (PI) which is defined as

12 $$\alpha ({\theta }^{\ast })=\frac{T-\mu ({\theta }^{\ast })}{\sigma ({\theta }^{\ast })},$$

where *T* is the target, *μ*(θ^*^) is the predicted mean, and *σ*(θ^*^) is the predicted standard deviation of *f*(θ^*^). The target, is generally defined as the best evaluated response on the real system; that is *T* = max(**f**), where, max(**f**) = max(**e** + **f**_**e**_), when using a model to inform the prior. Optionally, a value that diminishes with successive evaluations may be added to this. Alternatively, the target may be set to a known reachable value. Both the option of adding a successively diminishing value and defining the target as a known reachable value causes the acquisition function to be more explorative in selecting earlier evaluation steps.

**Algorithm 2:**
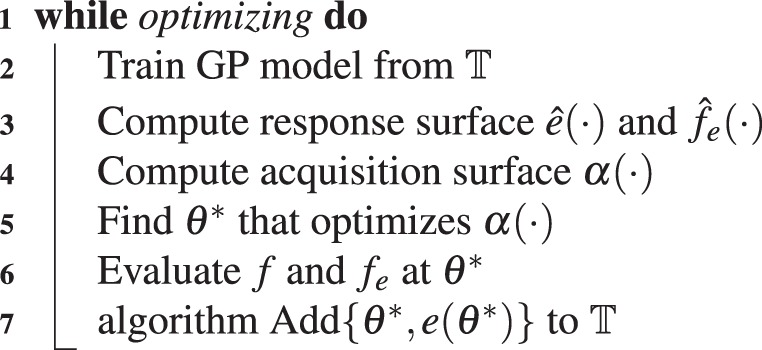
Modified algorithm.

## Results and Discussion

To validate the dynamics model of both the leg-body-body-leg and leg-body-leg-body-leg configurations of the crawling robot, test gaits were determined by first optimizing on the respective dynamics models in simulation. These gaits were then applied to the real robot and the performance compared to that of the simulations. These results show good agreement between the performance of the real crawling robot and its performance in simulation. The validated dynamics model for the leg-body-body-leg configuration was then used in simulation to compare the performance of Bayesian optimization to a finite difference policy gradient method to motivate the use of Bayesian optimization as a gait learning method. These simulations showed that Bayesian optimization outperformed a finite difference policy gradient method, supporting the hypothesis that Bayesian optimization will lead to convergence in fewer trials than a finite-difference policy search method making it more suitable for deployment on IPMC based robots in real-time. Bayesian optimization was then conducted on the real crawling robot, specifically to compare Bayesian optimization conducted from a uniform prior distribution and Bayesian optimization using a Gaussian process trained in simulation as the prior. Unfortunately, unexpected degradation of the IPMCs was observed with extended use. By the end of successive experiments the IPMC could not replicate its same level of performance upon application of the same crawling gait. This technically violates the assumption that successive evaluations would be independent and identically distributed (IID) and thus invalidates the use of a Gaussian process to model the objective function. In spite of this, both approaches to Bayesian optimization show improving performance with successive evaluations. This section will first discuss the simulation methods and results and then the experiment methods and results.

### Simulation results and discussion

Optimization conducted to determine the test gaits for both the leg-body-body-leg and leg-body-leg-body-leg configurations was done by running successive simulations on the dynamics models in Simulink using 10-minute runs (simulation-time), using automatic solver selection with a variable step size. The Gaussian process used as the prior for subsequent optimization on the real robot was also obtained from the simulation on the dynamics model of the leg-body-body-leg configuration.

Comparison of Bayesian optimization and the policy gradient method were conducted by running successive simulations on the dynamics model of the leg-body-body-leg configuration of the crawling robot in Simulink using 15-second runs (simulation-time), using automatic solver selection with a variable step size. A random variable was added to the simulated distance traveled to add noise to the results. The decision variables used for optimization were the phases of the inputs to the individual body and leg components. Each optimization method was tested under three different noise levels (no noise, a signal to noise ratio of 1 and a signal to noise ratio of 10). Two initial conditions were tested under each noise level. For each initial condition, two trials were run to account for randomness in the simulations. Since, an advantage of Bayesian optimization is its ability to encode a known reachable target value, the performance of both Bayesian optimization where the target is defined as the maximum of the training data and Bayesian optimization where the target is defined as 95% the maximum possible distance are tested.

Table 2 gives the optimized phases of the inputs for both the the leg-body-body-leg and the leg-body-leg-body-leg configurations. In this table, the bodies and legs are enumerated moving forward along the direction of travel. As can be seen from Table 2, Bayesian optimization for the leg-body-body-leg configuration results in converged gait phases of 0°, 0°, 270° and 90° for body 1, body 2, leg 1 and leg 3 respectively and Bayesian optimization for the leg-body-leg-body-leg configuration results in converged gait phases of 0°, 30°, 270°, 0° and 110° for body 1, body 2, leg 1, leg 2, and leg 3 respectively. The effect of adding the middle leg to the robot is to cause the maximum possible speed to decrease. Notably the converged gait for the leg-body-leg-body-leg configuration only has body 2 and leg 3 slightly lagging their converged phases in the leg-body-body-leg configuration.

**Table 2:** Optimized phases for Bayesian optimization in simulation.

	Body 1	Body 2	Leg 1	Leg 2	Leg 3
leg-body-body-leg	0°	0°	270°	n/a	90°
leg-body-leg-body-leg	0°	30°	270°	0°	110°

**Figure 6:**
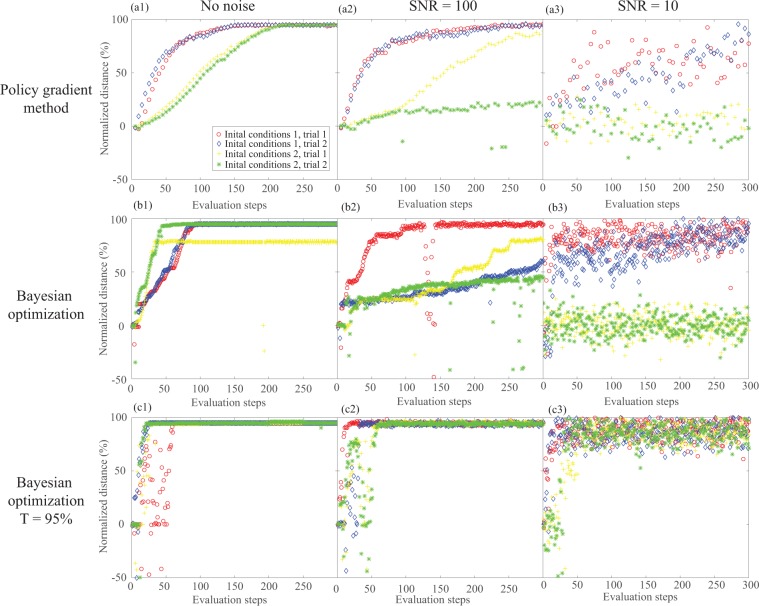
Policy gradient method using finite-difference gradient estimation vs. Bayesian optimization using probability of improvement (PI) acquisition function, with multiple trials from different ICs and signal to noise ratios (SNR)s: (**a1**) policy gradient with no noise, (**a2**) policy gradient with SNR = 100, (**a3**) policy gradient with SNR = 10, (**b1**) Bayesian optimization with target equal to maximum of training data with no noise, (**b2**) Bayesian optimization with target equal to maximum of training data with SNR = 100, **(b3**) Bayesian optimization with target equal to maximum of training data with SNR = 10, (**c1**) Bayesian optimization with target equal to 95% maximum possible distance with no noise, (**c2**) Bayesian optimization with target equal to 95% maximum possible distance with SNR = 100, (**c3**) Bayesian optimization with target equal to 95% maximum possible distance with SNR = 10.

Figure 6 compares the use of finite-difference policy gradient method^47^ and Bayesian optimization in simulation on the dynamics model of the leg-body-body-leg configuration of the crawling robot. Figure 6(a1–a3) shows the performance of the finite difference policy gradient method. Figure 6(b1–b3) shows the performance of Bayesian optimization using the probability of improvement acquisition function where the target is defined as the maximum of the training data. Figure 6(c1–c3) shows the performance of Bayesian optimization using the probability of improvement acquisition function where the target is defined as 95% the maximum possible distance of the robot. Bayesian optimization using both the maximum of the training data as the target and 95% the maximum possible distance as the target, perform as well or better than the policy gradient method. These results support the hypothesis that Bayesian optimization will be superior for use on the real crawling robot.

### Experiment results and discussion

Experiments on the crawling robot were conducted using Simulink real-time and Ni-Daq control hardware to output control signals and LM675T power amplifiers were used to power the signals. For comparison of the simulated and experimental performance of the crawling robot in response to the test gaits determined by optimizing in simulation, video recordings were taken and the Kanade-Lucas-Tomasi (KLT) algorithm was used to track points on the robots at the locations of the rear leg (*x*_1_), central leg or central joint (*x*_2_), and front leg (*x*_3_), respectively, in the videos of the experimental trials.

**Figure 7:**
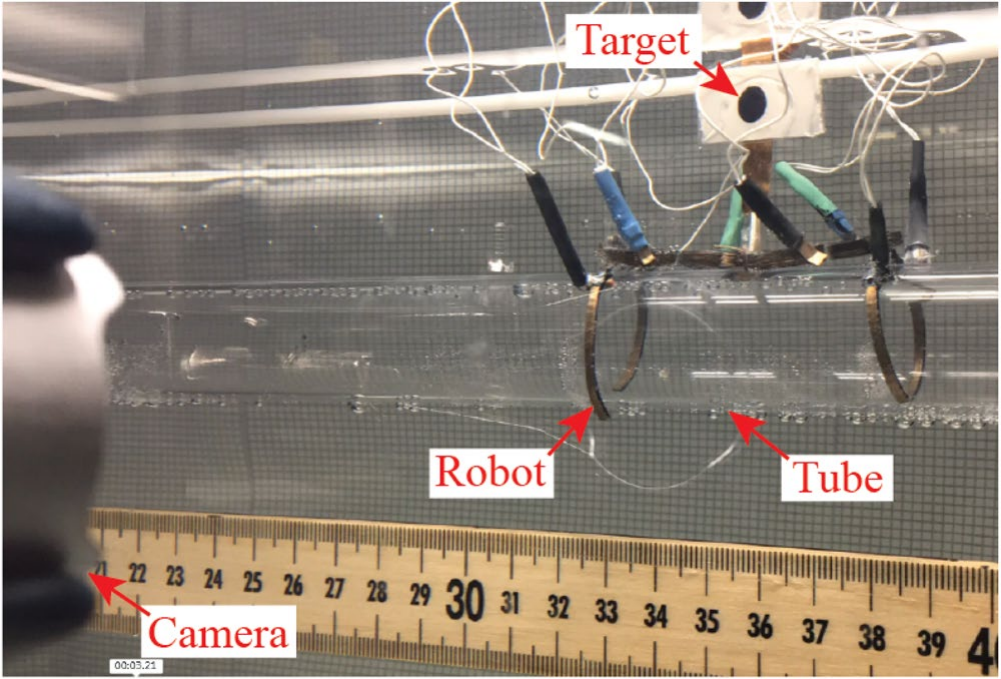
Experimental setup for optimization showing the robot, and the target and camera used to acquire the robot’s position. Optimization and control is conducted using Matlab and Simulink Real-Time software package.

To compare Bayesian optimization conducted starting from a uniform prior distribution to that of Bayesian optimization using Gaussian process trained in simulation as the prior, the leg-body-body-leg configuration was used and a webcam was added to track the robot’s position for 60 second evaluations of a set of gait parameters, as shown in Fig. 7. The direction of each trial was selected to automatically center the robot (*i.e*., if the robot was to the right of its origin at the beginning of a trial it would move left and vice-versa).

**Figure 8:**
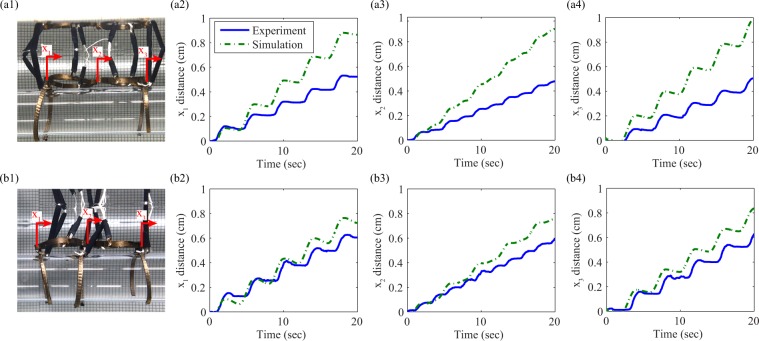
Experimental and simulated performance for optimized gaits for both the leg-body-body-leg and leg-body-leg-body-leg configurations showing the positions of the rear leg, the central joint and the front leg respectively as functions of time: (**a1**) leg-body-body-leg configuration of real robot, (**a2**) position of front leg of leg-body-body-leg configuration, (**a3**) position of central joint of leg-body-body-leg configuration, (**a4**) position of rear leg of leg-body-body-leg configuration, (**b1**) leg-body-leg-body-leg configuration of real robot, (**b2**) position of front leg of leg-body-leg-body-leg configuration, (**b3**) position of central leg of leg-body-leg-body-leg configuration and (**b4**) position of rear leg of leg-body-leg-body-leg configuration.

Figure 8 shows the experimental motion of the tracked locations for the leg-body-body-leg configuration and the leg-body-leg-body-leg configuration over 20 seconds compared to their simulated motion. Figure 8(a2,b2) show the motion of the rear legs, Fig. 8(a3,b3) show the motion of the central joint, and Fig. 8(a4,b4) show the motion of the front legs. The real speed of the leg-body-body-leg configuration with input phases of 0°, 0°, 270° and 90° for body 1, body 2, leg 1 and leg 2 respectively is 2.15 cm/min as compared to the simulated speed of 2.9 cm/min. The real speed of the leg-body-leg-body-leg configuration with input phases of 0°, 30°, 270°, 0° and 110° for body 1, body 2, leg 1, leg 2, and leg 3 respectively is 1.8 cm/min as compared to the simulated speed of 2.3 cm/min. Neglecting non-linear friction in the model is responsible for the discrepancies between the simulated and experimental performance. For instance, comparing the plots of the experimental and simulated motion in Fig. 8(b2), there are spans of time during which the velocity of the leg of the real robot is 0. By contrast, the velocity of the leg of the simulated robot is only momentarily zero. This difference is because the dynamics of crawling robot were characterized assuming a linear viscous friction effect. This neglects a nonlinear stiction effect by which there is a range of inputs for which the robot will not move, after coming to rest.

**Figure 9:**
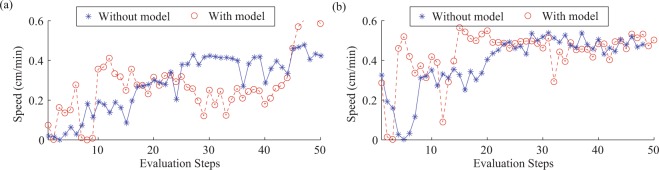
Speed for each evaluation step in Bayesian optimization on the real robot done both from a uniform prior (without a model) and using the GP trained in simulation as the prior (with a model): (**a**) in the leftwards direction (**b**) in the rightwards direction.

Figure 9 shows the results of Bayesian optimization on the crawling robot using a uniform prior distribution and of Bayesian optimization using a Gaussian process trained in simulation as the prior. Specifically, Fig. 9 shows the speed attained at each evaluation step for both optimization methods. Figure 9(a) shows these speeds in the leftwards direction and Fig. 9(b) shows the these speeds in rightwards direction. Neither the optimization from a uniform prior nor the optimization using a GP trained in simulation as the prior show clear convergence for the leftwards direction. The optimization from a uniform prior reaches a maximum speed of 0.5 cm/min and optimization using the GP trained in simulation as the prior reaches a maximum speed of about 0.6 cm/min. Both optimization from a uniform prior and using the GP trained in simulation as the prior converge to a speed of 0.5 cm/min for optimization in the rightwards direction. The optimization from a uniform prior converges after 20 evaluations and the optimization using the GP trained in simulation as the prior converges after 15 evaluations. Table 3 shows the final optimized gait phases for both the leftwards and rightwards directions. For the leftwards direction the optimization does not seem to converge but the final input phases were 0°, 40°, 100° and 250° for body 1, body 2, leg 1 and leg 2 respectively. For the rightwards direction the converged input phases were 0°, 0°, 240° and 100° for body 1, body 2, leg 1 and leg 2 respectively. The lower speeds compared with those achieved using test gaits derived from optimizing in simulation could be due to the degradation of the IPMC performance with successive evaluations. This would also effect the optimization algorithm, possibly preventing it from converging to the optimum gait. As a consequence of the degradation of the IPMC actuators performance over time, the objective function being optimized over would in fact be time dependent. This would invalidate the assumption implicit in a Gaussian process, that successive evaluations of the same parameter set would be from identical distributions. This problem could possibly be ameliorated by using different policy representations and ensemble methods in the future.

**Table 3:** Optimized phases for Bayesian optimization on robot.

	Body 1	Body 2	Leg 1	Leg 2
Leftwards	0	40°	100°	250°
Rightwards	0	0°	240°	100°

## Emerging Challenges

In the development of the 3D printing and learning-based control of IPMC actuators, several challenges have emerged that can be addressed by future research. One of the emerging challenges specific to 3D printing IPMCs is that increased printing resolution leads to increased failures in the printing process. This impedes the printing of thin-walled actuators that exhibit large displacements when actuated. This can potentially be ameliorated by a thorough study of the printing process variables. Additionally, alternative melt-processing-based 3D printing methods for Nafion precursor such as selective laser sintering (SLS) or use of alternative precursor materials, such as polystyrene in the FDM 3D printing process could also lead to increased resolution. In the use of Nafion precursor in an SLS 3D printing process, the critical inquiries would be into how the powder should be prepared and the required print settings for fabricating solid fused structures in Nafion precursor with high-resolution features, such as walls as thin as 0.1 mm or less. Alternative materials, such as Polystyrene, which are more easily printed than Nafion precursor through the FDM process, might also be considered. Materials such as polystyrene are more controllable in FDM 3D printing, because they are more rigid in their solid state, and have a lower melting temperature. However, a critical research consideration in adapting alternative materials to the print then functionalize paradigm is that these materials require *in situ* functionalization processes. In the case of polystyrene, for instance, this material requires identification of an *in situ* partial sulfonation process to obtain the functional material, lightly-sulfonated polystyrene, which acts as an ion-exchange membrane.

Another challenge to the 3D printing of IPMCs is control of electrode development in the post printing process, such that discrete electrode regions are generated without need for segmentation. This is especially needed for the manufacturing of complex multi-input-multi-output IPMC devices and systems, which require multiple electrode pairs. A potential solution to this challenge is incorporating a secondary material in the printing process, specifically to control electrode development in the post-printing steps. Since electrode development using reduction processes, such as are used for developing platinum on Nafion membranes, can only deposit platinum ions in functional materials, secondary materials can prevent the development of electrodes in selected regions by masking the ion-exchange material. Alternatively, another approach might be to print electrode materials directly onto the precursor or functionalized material.

In the area of motion control, performance degradation, especially when operated above the electrolysis voltage of the hydrating solvent^84^, is a significant emerging challenge to the control of IPMCs. This performance degradation is not well understood and can lead to significant under performance if performance degradation is not planned for or avoided. Since performance degradation changes the dynamic behavior of IPMCs over time, it invalidates control policies trained on an IPMC’s past performance. This is a challenge for conventional Bayesian optimization which treats each data point as drawn from an identical distribution. Moreover, since conventional control methods do not optimize the performance of a device over its functional lifetime, these methods could lead to accelerated degradation. To address this challenge, control techniques are needed that anticipate and plan for performance degradation. One possible approach is use of Gaussian processes to project IPMC performance over time, based on general trends for similar IPMCs and real-time degradation data of the IPMC being controlled.

## Conclusions

This paper described 3D printing for manufacturing and machine learning for control of example IPMC actuator components. A prototype modular reconfigurable soft crawling robot platform was described. The development of the platform was presented to illustrate the proof-of-concept of 3D-printing of IPMCs actuators and the application of machine learning for effective motion control. Furthermore, this paper tested two hypotheses: (1) that Bayesian optimization will lead to convergence in fewer trials than a finite-difference policy gradient method making it more suitable and practical for controlling IPMCs; and (2) that prior knowledge from a dynamics model (especially a known achievable target value) will lead to convergence in fewer number of trials than simply optimizing from a uniform prior distribution. To investigate these hypotheses, the dynamics of the crawling robot were modeled and the model was used to test various learning-based control methods in simulation. Bayesian optimization was also applied directly to the crawling robot to investigate the practical impact of the degradation on this motion control strategy. Simulation results indicate that Bayesian optimization employing prior knowledge in the form of a known achievable performance target does lead to much faster convergence than either Bayesian optimization without this knowledge or a conventional finite difference policy gradient method. Experiments directly on the crawling robot indicated that performance degradation is significant (reducing the crawling robot performance to less than a quarter of its initial achievable performance over the duration of optimization). This invalidates the performance level explicitly incorporated as the target in the acquisition function for Bayesian optimization. Technically, this would also invalidate the assumption implicit in the manner in which the GP was used, that successive evaluations of the same parameter set would be from identical distributions. None-the-less, Bayesian optimization still demonstrated continuous performance improvement with successive trials on the crawling robot. These results also established proof-of-concept for continued advancement of more complex IPMC devices.
